# Deep analysis of Loop L1 HVRs1-4 region of the hexon gene of adenovirus field strains isolated in Poland

**DOI:** 10.1371/journal.pone.0207668

**Published:** 2018-11-27

**Authors:** Jowita Samanta Niczyporuk

**Affiliations:** Department of Poultry Viral Diseases, National Veterinary Research Institute, Pulawy, Poland; Sechenov First Medical University, RUSSIAN FEDERATION

## Abstract

**Background:**

To date, studies on loop L1 HVRs1-4 region of the hexon gene in fowl adenovirus genome (FAdVs) lack comprehensive molecular data. In this study detailed prospectively obtained sequences from field adenovirus strains, NVRI, Poland have been analyzed.

**Methods:**

Overall hundred and thirty seven adenovirus strains were collected, evaluated, and examined of hyper variable loop L1 region HVRs1-4 of the hexon gene for the presence of similarity, mutations, tertiary structure, and spinal conformation.

**Results:**

Sequences were characterized, and divided for five species and seven types, FAdV-A-E/FAdV-1/2/4/5/7/8a/8b/11. The presence of predicted tertiary structure depending on type/species were determined. Analysis of specific selected sequences: GQMTN 1/A, 7/E, and 8b/E, GQMTT 2/11/D, GQLSN 4/C, GQMTH 5/B, and GQMSN 8a/E in examined HVRs1-4 Loop L1 region of hexon gene compared to tertiary structure indicated that this visibly conservative region represents the antigenic binging activity.

**Conclusion:**

This is the first molecular study on tertiary structure on HVRs1-4 region in adenovirus genome conducted in Poland. Analysis indicated specific sequence in Loop L1 HVR1-4 region which is strictly responsible for antibodies binding. This information could assist during the process connected with specific preventive strategies based on their molecular genome investigation and new facilitate studies. This study will help to better understand the mechanisms of pathogenicity of adenovirus strains provide a guide for disease control in birds.

## Introduction

**Fig 1:**
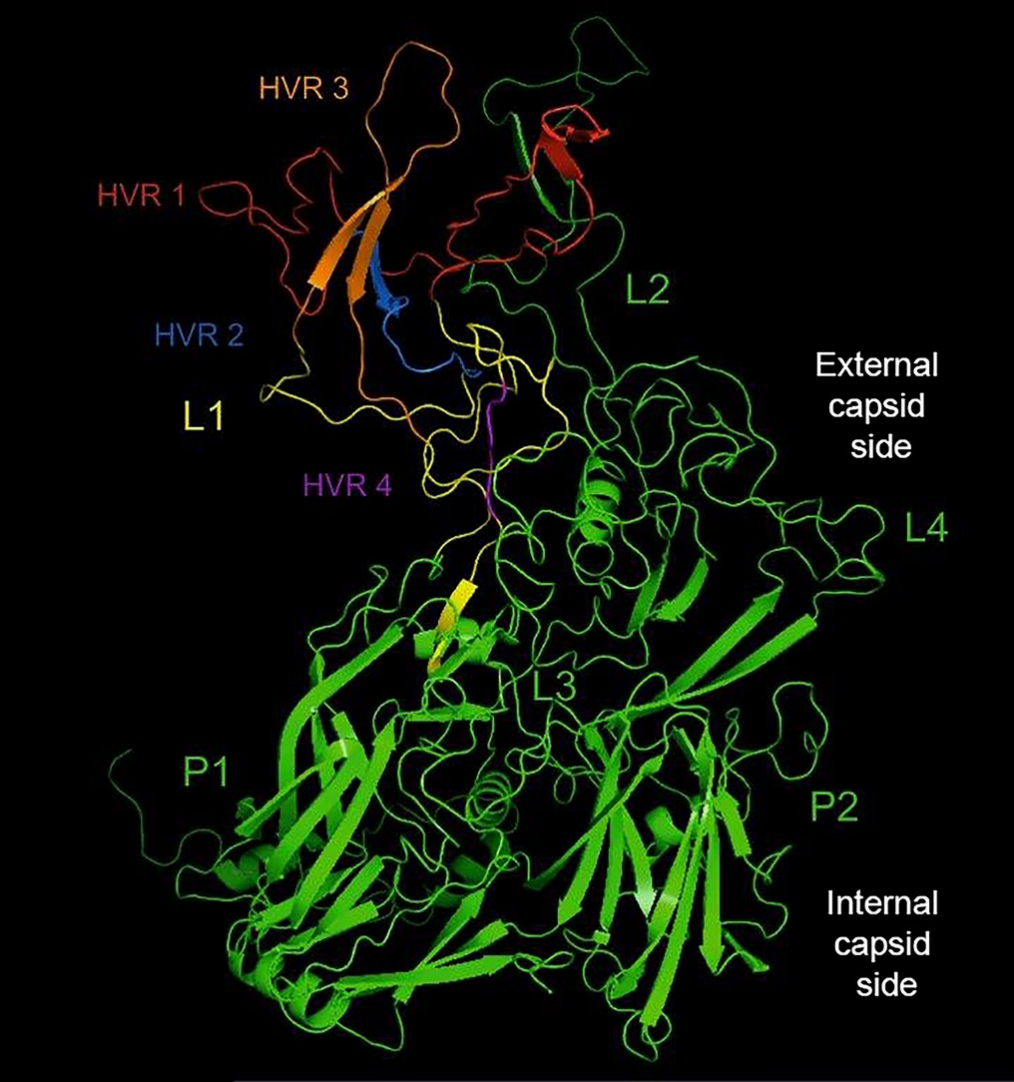
Triangular structure of adenovirus hexon protein. In colors indicated separate regions HVR1-4 of Loop L1 (NCBI)/CnD3 4.3 additionally divided into sections representing hyper variable regions HVR1-4, and regions P1 and P2 conservative.

Data connected with adenovirus structure, different protein functions, genome organization, and replication are based mostly on human adenovirus research [1]. The adenovirus capsid is formed by three major protein: hexon, penton base, and fiber, however the hexon, play an important role in the genome organization. Predicted amino acid sequences in hexon gene from human Ad2 and Ad5, and bovine adenovirus type 3 presented regions with high homology at the end of N and C regions, separated by central part with low homology. Both hexon protein have a common architecture. Hexon is the major capsid protein presented from 40 to 820 copies per virion. Every hexon has two characteristic parts: triangular peak with three towers, and base with central part. To construct from conservative component: regions P1 and P2 which are basement created of the particle and taking part in trimer formation. Regions hyperviriable HVR of hexon protein creates loops L1, L2, and L4, which are in external part of virion towers created (Fig 1). We can identify seven hyperviriable regions HVRs1-7 [2]. Four of them are located in Loop L1, two HVRs in Loop L2, and one HVR in Loop L4. Type specific antigenic determinants are coded by HVRs from loop L1 and L2, existed on the hexon surface, and are strictly responsible for induction of immunological response. In Loop L3 there are no such determinants [3]. Lenong, 2016 [4] indicated that three structure of Ad type2 (Ad2) of species CE3-19K/HLA-A2 complex showed the adaptation of a novel tertiary fold, and uses a new binding surface on HLA-A2. The adenoviruses encoded E3-19K immunomodulatory protein targets MHC class I molecules for retention within the endoplasmic reticulum (ER). Lenong, 2016 reported the x-ray crystal structure of the Ad type4 E3-19K of species E bound to HLA-A2 at 2.64 A resolution, and structural analysis shows that Ad4 E3-19K adopts a tertiary fold shared with Ad2 E3-19K/C. The authors reveals common binding characteristics that explain the promiscuous, and yet high-affinity, association of e3-19K proteins with HLA-A and -B molecules. Study of Lenong, 2016 provides new information into how E3-19K proteins selectively engaged with MHC I antigen presentation, and counteract activation of T-cells.

Significant differences in genome size may have an influence on the genome organisation [5, 6, 7]. The genome of avian adenoviruses is about 44–45 kb long, depending on the species/types. For comparison adenovirus strains from the genus *Siadenovirus* has the genome 26 kb long, genus *Mastadenovirus* has 31–36 kb, and the size of genus *Atadenovirus* genome is about 27.7 kb [1, 4, 8, 9, 10]. The shape of the trimeric hexon is not common, and is divided into a hexagonal base which is reach of β-structure, and a top triangular containing secondary structure [11]. In this study investigated for the first time in Poland the presence of tertiary structure and spinal conformation of hexon protein region Loop L1 HVRs1-4 in examined strains. Schematic concept of the structure of adenovirus genome, localisation of hexon gene, and HVRs1-4 was presented in Fig 1.

## Materials and methods

### Virus isolation

137 adenovirus strains were isolated from the gizzard, intestines, and liver of infected birds in which postmortem examination revealed changes in the liver, gizzards, and intestines characteristic for Inclusion Body Hepatitis (IBH), and Gizzard Erosion and Ulceration (GEU).

### DNA extraction

The DNA of 137 FAdV strains was extracted directly from internal organs of sick chickens by using a QIAamp mini-kit (Qiagen, Germany) according to manufacturer’s instructions. The negative DNA controls were extracted from non-infected CEFs. The DNA was stored at -20°C for the next step of the study, as the template for sequencing. Extracted FAdVs DNA were tested by PCR to confirm the absence of others pathogens as avian reovirus (ARV), chicken anemia virus (CAV), infectious bursa disease virus (IBDV), Marek’s disease (MD), and plaque passage have been done.

### Amplification of a target gene by PCR

The primers, FAdVF JSN (5’aatgtcacnaccgaraaggc3’) and FAdVR JSN (5’cbgcbtrcatgtactggta 3’) were used for PCR. They were designed using Primer 3 software (http://bioinfo.ut.ee/primer3-0.4.0/) and were based on a loop L1 HVR1-4 fragment of the hexon gene that is strictly conserved among FAdV types, at nucleotide position 178–1017 (based on FAdV-1, GenBank accession number AC_000014).

PCR was conducted in a final volume of 25 μL of reaction mixture, which contained 2.5 μL of 10x PCR buffer, 1 μL of dNTPs (10 mM) (Fermentas, USA), 1.5 μL of each of the primers (10 μM), 2 μL of the DNA template, 11.5 μL of sterile water, and 1.0 μL of DNA polymerase. After pre-denaturation at 95 °C for five min, the extract was denatured at 94 °C for 45 s, and the primer was annealed at 55 °C for 1 min, followed by product elongation at 72 °C for 2 min and a final elongation at 72 °C for 10 min. Thirty-five amplification cycles were performed using a basic gradient thermocycler (Biometra, Germany). The results were considered positive if the resulting DNA product had the predicted size of 830 bp. After the amplification, PCR products were purified using a NucleoSpin Extract II Kit (Macherey-Nagel, France) and sent to GenoMed (Warsaw) for sequencing.

### Sequence alignment and phylogenetic analysis

Sequencing was performed using the Sanger method and a GS FLX/Titanium sequencer (Roche, Switzerland) at Genomed (Warsaw). The sequences obtained for the loop L1 HVRs1-4 region of the hexon gene of the 137 adenovirus strains respectively have been determined. The level of nucleotide and amino acid sequence identity and the type/species designation have been determined. Sequences of five reference adenovirus species A-E were also obtained from the GenBank database, and molecular analysis was performed using the computer software MEGA7, Geneious 7.2, and BLAST.

### Adenovirus field strain sequences

137 sequences of HVRs1-4 loop L1 region of hexon gene have been used for molecular study.

### Adenovirus reference strain sequences

The full-length sequence of reference strains, belonging to the type/species FAdV-2/11, 4, 5, 7, 8a, and 8b derived from the GenBank database (NCBI) were used for nucleotide and amino acid sequence comparisons.

### Consensus HVRs1-4 sequences created

For the presence and indication of the structure of 137 adenovirus strains, sequences HVRs1-4 Loop L1 of the hexon gene have been prepared. The consensus sequences were prepared by aligning DNA sequences of strains classified to one type. This way seven consensus sequences representing seven types and five species were received. During the confirmation process of the correctness of created consensus sequences, analysis of similarity with reference and field sequences of adenovirus strains was conducted. The annealing specific for each type have been created and consensus sequences specific for seven types were formed. The analysis were performed with Geneious7.2 software.

### Analysis of nucleotide sequences

The percentage of similarity between the examined field and reference adenovirus strain sequences HVRs1-4 loop L1 was determined by using Geneiouse7.2 software. During comparisons, the most common nucleotides in same positions in examined region of hexon gene in strains belonging to seven different types were determined.

### Determinations of mutations

The influence of each single mutation on the nucleotide, and amino acid sequence in examined region of Loop L1 HVRs1-4 hexon gene was analysed. During the study, the consensus nucleotide sequences of the examined strains/types were translated in order to describe differences in amino acid sequences. Seven amino acid sequences between169 aa to 177 aa long, were obtained.

### Tertiary structure designation

The next step of the study determination of the tertiary structure (spatial conformation) have been performed. The occurrence of predicted structure in HVRs1-4 Loop L1 region were compared with the model of protein hexon 3D structure of adenovirus hexon trimer from reference strain FAdV1/A CELO (GenBank Accession Number AF339914) from the NCBI database. Analysis of theoretic tertiary structures of hexon protein performed by using Geneiouse7.2 software, and submitted data from database GenBank (NCBI)/CnD3 4.3.

## Results and discussion

The presence of the genetic material of field adenovirus strains was found in the internal organs of 137 sick chickens sent to NVRI, Department of Poultry Disease for diagnostic examinations. The chickens have liver inflammation and gizzards erosions with clinical manifestations in the digestive tract. After the examinations FAdVs infection was confirmed. The information on virus, and year isolation, clinical signs of birds, birds type, age, and other information have been presented in Table 1.

**Table 1:** Overview of collected adenovirus field strains at the Department of Poultry Diseases, NVRI, Poland.

Number	Virus isolation	Year of isolation	Clinical signs of birds	Pultry type	type/species
1	23/07	2007	Nonsymptomatic	Layer 16 weeks	FAdV-7/E
2	45/09	2009	Nonsymptomatic	Layer 18 weeks	FAdV-7/E
3	50/03	2003	Nonsymptomatic	Layer 22 weeks	FAdV-7/E
4	17/09	2009	Sadness	Broiler 4.5 weeks	FAdV-7/E
5	72/08	2008	Nonsymptomatic	Layer 39 weeks	FAdV-7/E
6	19-8/10wF	2010	Depressed	Layer 24 weeks	FAdV-7/E
7	15-14-10kij	2010	IBH clinical case	Broiler 19 weeks	FAdV-7/E
8	14-7-10j5F	2010	Depressed	Broiler 29 weeks	FAdV-7/E
9	13-7-10z4F	2010	IBH clinical case	Broiler 29 weeks	FAdV-7/E
10	12-7-10w4F	2010	IBH clinical case	Broiler 29 weeks	FAdV-7/E
11	11-6-10wF	2010	Crouching position	Layer 9 weeks	FAdV-7/E
12	10-5-10jF	2010	Nonsymptomatic	Layer 17 weeks	FAdV-7/E
13	9-5-10wF	2010	Nonsymptomatic	Layer 17 weeks	FAdV-7/E
14	8-2-10F	2010	IBH clinical case	Broiler 6 weeks	FAdV-7/E
15	4-82-09F	2009	Depressed	Layer 25 weeks	FAdV-7/E
16	12/10j	2010	Depressed	Layer 37 weeks	FAdV-7/E
17	11/10j	2010	Depressed	Layer 25 weeks	FAdV-7/E
18	36/09	2009	Nonsymptomatic	Layer 17 weeks	FAdV-7/E
19	82/09w	2010	Depressed	Layer 33 weeks	FAdV-7/E
20	35/08	2008	IBH clinical case	Broiler 12 days	FAdV-7/E
21	20-8-10jF	2010	Nonsymptomatic	Layer 24 weeks	FAdV-7/E
22	20-8-10jF2	2010	Nonsymptomatic	Layer 24 weeks	FAdV-7/E
23	34/08	2008	IBH clinical case	Broiler 12weeks	FAdV-7/E
24	8/10w	2008	IBH clinical case	Broiler 7 weeks	FAdV-7/E
25	36/09s	2007	Depressed	Layer 27 weeks	FAdV-7/E
26	47/09	2009	IBH clinical case	Broiler 7 weeks	FAdV-7/E
27	14/10w	2010	Depressed	Layer 12 weeks	FAdV-7/E
28	5-81-09F	2009	Nonsymptomatic	Layer 26 weeks	FAdV-8a/E
29	5-81-09F(2)	2009	Nonsymptomatic	Layer 26 weeks	FAdV-8a/E
30	51/04	2004	Nonsymptomatic	Layer 27 weeks	FAdV-8a/E
31	96/10j	2010	IBH clinical case	Broiler 6 weeks	FAdV-8a/E
32	17/10jF	2010	Nonsymptomatic	Layer 20 weeks	FAdV-8a/E
33	38-10R	2010	Nonsymptomatic	Layer 38 weeks	FAdV-8a/E
34	7-1-10F	2010	IBH clinical case	Broiler 3 weeks	FAdV-8a/E
35	37/10z	2010	IBH clinical case	Broiler 8 weeks	FAdV-8a/E
36	6-86-09BF	2009	Digestive system dysfunction	Layer 28 weeks	FAdV-8a/E
37	35/09	2009	IBH clinical case	Broiler 12 days	FAdV-8a/E
38	48/08	2008	IBH clinical case	Broiler 8 days	FAdV-8a/E
39	8/08	2008	Digestive system dysfunction	Layer 26 weeks	FAdV-8a/E
40	94/10j	2010	Digestive system dysfunction	Layer 12 weeks	FAdV-8a/E
41	94/10z	2010	Nonsymptomatic	Layer 12 weeks	FAdV-8a/E
42	46/10jR	2010	IBH clinical case	Broiler 5 weeks	FAdV-8a/E
43	24/12z	2012	Nonsymptomatic	Layer 12 weeks	FAdV-8a/E
44	81/09w	2009	Nonsymptomatic	Broiler 8 weeks	FAdV-8a/E
45	26/11j	2011	IBH clinical case	Layer 12 weeks	FAdV-8a/E
46	26/11z	2011	IBH clinical case	Broiler 8 weeks	FAdV-8a/E
47	13-12/z	2012	IBH clinical case	Layer 12 weeks	FAdV-8a/E
48	6/12j	2012	IBH clinical case	Broiler 6 weeks	FAdV-8a/E
49	75/08	2008	IBH clinical case	Broiler 7 weeks	FAdV-8b/E
50	32/07	2007	Depressed	Layer 27 weeks	FAdV-8b/E
51	60/10z	2010	Depressed	Layer 12 weeks	FAdV-8b/E
52	102/10j	2010	Digestive system dysfunction	Layer 35 weeks	FAdV-8b/E
53	58/11w	2011	Nonsymptomatic	Broiler 6 weeks	FAdV-8b/E
54	35/11j	2011	Nonsymptomatic	Broiler 8 weeks	FAdV-8b/E
55	52/11z	2011	Nonsymptomatic	Broiler 7 weeks	FAdV-8b/E
56	10/10jF	2010	Nonsymptomatic	Broiler 6 weeks	FAdV-8b/E
57	16-14-10k2	2010	IBH clinical case	Broiler 29 days	FAdV-8b/E
58	16-14-10k2F	2010	IBH clinical case	Broiler 19 weeks	FAdV-8b/E
59	1-66-09F	2009	IBH clinical case	Broiler 6,5 weeks	FAdV-1/A
60	27/10jF	2010	Nonsymptomatic	Layer 14 weeks	FAdV-1/A
61	110/10z	2010	IBH clinical case	Broiler 2 days	FAdV-1/A
62	14/08	2008	Nonsymptomatic	Layer 27 weeks	FAdV-1/A
63	56/11z	2011	Nonsymptomatic	Broiler 6 weeks	FAdV-1/A
64	61/11z	2011	Nonsymptomatic	Broiler 6 weeks	FAdV-1/A
65	35/10w	2010	IBH clinical case	Broiler 2 weeks	FAdV-4/C
66	62/10z	2010	Nonsymptomatic	Layer 12 weeks	FAdV-4/C
67	31/10z	2010	Nonsymptomatic	Layer 27 weeks	FAdV-4/C
68	64/10j	2010	Nonsymptomatic	Layer 14 weeks	FAdV-4/C
69	131-10Tj	2010	IBH clinical case	Broiler 5 weeks	FAdV-5/B
70	127-10TF	2010	IBH clinical case	Broiler 5 weeks	FAdV-5/B
71	126/10j	2010	Nonsymptomatic	Broiler 5 weeks	FAdV-5/B
72	88/10z	2010	Nonsymptomatic	Broiler 6 weeks	FAdV-5/B
73	88/10j	2010	Nonsymptomatic	Broiler 6 weeks	FAdV-5/B
74	21-133-10j	2010	IBH clinical case	Broiler 6 weeks	FAdV-5/B
75	45/10jR	2010	IBH clinical case	Broiler 4,5 weeks	FAdV-5/B
76	131/10TF	2010	IBH clinical case	Broiler 2 weeks	FAdV-5/B
77	45/11z	2011	Nonsymptomatic	Broiler 6 weeks	FAdV-5/B
78	88/11z	2011	Nonsymptomatic	Broiler 2 weeks	FAdV-5/B
79	86/11j	2011	Nonsymptomatic	Broiler 8 weeks	FAdV-5/B
80	55/11w	2011	Nonsymptomatic	Broiler 8 weeks	FAdV-5/B
81	3/12z	2012	Nonsymptomatic	Broiler 2 weeks	FAdV-5/B
82	119/10j	2010	Depressed	Layer 33 weeks	FAdV-2/11/D
83	22/10wF	2010	IBH clinical case	Broiler 2,5 weeks	FAdV-2/11/D
84	46/09	2009	Nonsymptomatic	Layer 49 weeks	FAdV-2/11/D
85	32/10j	2010	Nonsymptomatic	Layer 16 weeks	FAdV-2/11/D
86	18-15-10jF	2010	IBH clinical case	Broiler 4,5 weeks	FAdV-2/11/D
87	121/10j	2010	Nonsymptomatic	Layer 17 weeks	FAdV-2/11/D
88	117/10j	2010	Digestive system dysfunction	Broiler 6,5 weeks	FAdV-2/11/D
89	24-6-11w	2011	Nonsymptomatic	Layer 3 weeks	FAdV-2/11/D
90	22-4-11w	2011	Nonsymptomatic	Layer 20 weeks	FAdV-2/11/D
91	54-10w3	2010	IBH clinical case	Broiler 4 weeks	FAdV-2/11/D
92	25/09	2009	Nonsymptomatic	Layer 29 weeks	FAdV-2/11/D
93	Imielno	2003	Nonsymptomatic	Layer 18 weeks	FAdV-2/11/D
94	17-15-10jF6	2010	Digestive system dysfunction	Broiler 4,5 weeks	FAdV-2/11/D
95	2-68-09	2009	IBH clinical case	Broiler 7 weeks	FAdV-2/11/D
96	42/10wR	2010	IBH clinical case	Broiler 21 days	FAdV-2/11/D
97	86/09j	2009	Nonsymptomatic	Layer 28 weeks	FAdV-2/11/D
98	49-10F-6-	2010	Nonsymptomatic	Broiler 6 weeks	FAdV-2/11/D
99	38-10(2)	2010	Nonsymptomatic	Layer 38 weeks	FAdV-2/11/D
100	105-10TF	2010	Nonsymptomatic	Broiler 8 weeks	FAdV-2/11/D
101	87/10j	2010	IBH clinical case	Broiler 6,5 weeks	FAdV-2/11/D
102	87/10z	2010	IBH clinical case	Broiler 6,5 weeks	FAdV-2/11/D
103	25-119-10guz-w	2010	Nonsymptomatic	Layer 33 weeks	FAdV-2/11/D
104	65/10w	2010	IBH clinical case	Broiler 3 weeks	FAdV-2/11/D
105	66-10-(1–60)	2010	IBH clinical case	Broiler 2 weeks	FAdV-2/11/D
106	67-10zoltko	2010	Nonsymptomatic	Layer 17 weeks	FAdV-2/11/D
107	69/10w	2010	IBH clinical case	Broiler 4,5 weeks	FAdV-2/11/D
108	70/10z	2010	Nonsymptomatic	Layer 14 weeks	FAdV-2/11/D
109	74/10j	2010	Nonsymptomatic	Layer 14 weeks	FAdV-2/11/D
110	75/10z	2010	IBH clinical case	Broiler 8 days	FAdV-2/11/D
111	49-10N	2010	IBH clinical case	Broiler 6 weeks	FAdV-2/11/D
112	29/07	2007	Nonsymptomatic	Layer 27 weeks	FAdV-2/11/D
113	19/10j	2010	Dead birds	Broiler 8 days	FAdV-2/11/D
114	20/10F	2010	Dead birds	Broiler 13 days	FAdV-2/11/D
115	21-10F1	2010	IBH clinical case	Broiler 2 weeks	FAdV-2/11/D
116	21-10F2	2010	IBH clinical case	Broiler 2 weeks	FAdV-2/11/D
117	69/11z	2011	Nonsymptomatic	Broiler 8 weeks	FAdV-2/11/D
118	67/11z	2011	Nonsymptomatic	Broiler 8 weeks	FAdV-2/11/D
119	62/11z	2011	Nonsymptomatic	Broiler 8 weeks	FAdV-2/11/D
120	5/11w	2011	Nonsymptomatic	Layer 12 weeks	FAdV-2/11/D
121	46/11w	2011	Nonsymptomatic	Layer 25 weeks	FAdV-2/11/D
122	46/11j	2011	Nonsymptomatic	Layer 25 weeks	FAdV-2/11/D
123	38/12z	2012	IBH clinical case	Broiler 2 weeks	FAdV-2/11/D
124	36/11w	2011	IBH clinical case	Broiler 2 weeks	FAdV-2/11/D
125	34/11z	2011	IBH clinical case	Broiler 3 weeks	FAdV-2/11/D
126	33/11w	2011	Nonsymptomatic	Layer 27 weeks	FAdV-2/11/D
127	33/11j	2011	Nonsymptomatic	Layer 27 weeks	FAdV-2/11/D
128	25/09w	2009	IBH clinical case	Broiler 2 weeks	FAdV-2/11/D
129	23/12z	2012	IBH clinical case	Broiler 2 weeks	FAdV-2/11/D
130	57/11j	2011	IBH clinical case	Broiler 2 weeks	FAdV-2/11/D
131	43/10w	2010	IBH clinical case	Broiler 3 weeks	FAdV-2/11/D
132	20/11j	2011	Nonsymptomatic	Layer 20 weeks	FAdV-2/11/D
133	20/11z	2011	Nonsymptomatic	Layer 20 weeks	FAdV-2/11/D
134	12/11j	2011	Digestive system dysfunction	Broiler 4,5 weeks	FAdV-2/11/D
135	12/11z	2011	Digestive system dysfunction	Broiler 4,5 weeks	FAdV-2/11/D
136	43/10j	2010	Nonsymptomatic	Broiler 8 weeks	FAdV-2/11/D
137	57/11w	2011	Nonsymptomatic	Broiler 8 weeks	FAdV-2/11/D

Under molecular examinations the 137 nucleotide sequences were divided, and obtained strain sequences constructed five main branches representing species from FAdV-A to E. Branch FAdV-E formed three subbranches represented by types; FAdV-7, FAdV-8a, and FAdV-8b. On this basis, adenovirus strains were grouped into five species, which were represented by eight types: FAdV-1, FAdV-2, FAdV-11, FAdV-4, FAdV-5, FAdV-7, FAdV-8a, and FAdV-8b. The biggest group was formed by the 56 strains classified as type 2/11/D, and the smallest group by type FAdV-4/C was formed by four strains under the sequencing examinations. Each of them were compared to a selected reference strains: AC_000014/1/A, KT862805/KT862812/2/11/D, HE608152/4/C, KC493646/5/B, KT862809/7/E, KT862810/8a/E, KT862811/8b/E), respectively.

With the purpose of confirmation of accuracy analysis, created consensus sequences of examined strains were compared to the reference strain sequences. The percentage of similarity between consensus sequences for examined seven types with region HVRs1-4 of Loop L1 hexon gene and reference strains were presented in Table 2. In this way, the model sequence representative for seven examined adenovirus types was created: FAdV-1/A from six strains, FAdV-2/11/D from 56 strains, FAdV-4/C from four strains, FAdV-5/B from thirteen strains, FAdV-7/E from 27 strains, FAdV-8a/E from 21 strains, and FAdV-8b/E from ten strains, and in the next step of the study they were the base for all analysis.

**Table 2:** Percentage of similarity between the field and reference adenovirus strains sequence HVRs1-4 Loop L1. Field strains indicated as: FAdV-1/A, FAdV-2/11/D, FAdV-4/C FAdV-5/B FAdV-7/E FAdV-8a/E FAdV-8b/E, and reference strains as: FAdV-1/A AC000014, FAdV2/11/D-KT862805, FAdV-4/C HE608152, FAdV-5/B KC493646, FAdV-7/E KT862809, FAdV-8a/E KT862810, FAdV-8b/E KT862811.

Type %	FAdV-1/A	FAdV-2/11/D	FAdV-4/C	FAdV-5/B	FAdV-7/E	FAdV-8a/E	FAdV-8b/E
FAdV-1/A AC000014	X	56,6	63,9	59,9	59,3	58,1	56,1
FAdV-2/11/D KT862805	56,6	X	51,2	63,5	67,2	70,1	67,0
FAdV-4/C HE608152	63,9	51,2	X	56,4	55,4	55,8	53,5
FAdV-5/B KC493646	59,9	63,5	56,4	X	64,8	65,9	62,7
FAdV-7/E KT862809	59,3	67,2	55,4	64,8	X	81,5	86,7
FAdV-8a/E KT862810	58,1	70,1	55,8	65,9	81,5	X	78,1
FAdV-8b/E KT862811	56,1	67,0	53,5	62,7	86,7	78,1	X

Seven consensus sequences have been created, and most numerous mutations in the first and second place of codon were detected in strains belongs to type FAdV-4/C (97) with 61% of all mutations in that region. The lowest quantity 33 (40%) of substitutions was found in type FAdV-8b/E. Average number of substitutions influence on amino acid coding and was (53.6) what was 47%. Data describing this analysis are presented in Table 3. The lowest percentage of the similarity was obtained between strain sequences from type/species FAdV-2/11/D, and sequences derived from strains representing type FAdV-4/C. This percentage was (51.2%). The highest similarity (86.7%) was between strains representing type FAdV-7/E, and FAdV-8b/E. Substitutions were most frequent in the examined region of type FAdV-4/C and were estimated at 158. The lowest number of substitutions 71 was found in type FAdV-7/E. Average value for all types was 113.7. Many of substitutions in nucleotide sequences had an impact on the kind of amino acid coding [12]. The quantity of such substitutions on the first or second place in codon was also determined (data was not published yet).

**Table 3:** Mutations in the Loop L1 region of the hexon gene in the examined HVRs1-4.

type	Quantity of mutations	Quantity of nonsynonymous mutations	%
FAdV-1/A AC_000014	138	73	53
FAdV-2/11/D KT862805 KT862812	131	43	33
FAdV-4/C HE608152	158	97	61
FAdV-5/B KC493646	135	66	49
FAdV-7/E KT862809	71	28	39
FAdV-8a/E KT862810	80	35	44
FAdV-8b/E KT862811	83	33	40
	113,71	53,57	47

Table 3% is the ratio of nonsynonymous mutations

Four hyper variable regions HVRs1-4 in Loop L1 were defined by the searching of conservative and variable sequences (Fig 1). Altogether four HVRs covered region of approximately 122 aa, depending on type/species. Regions with the highest variability, adequate of HVR1-4 region of Loop L1 and the position of nucleotides and amino acids, were presented in Table 4. To indicate more significant differences, the similarities between the sequences in HVRs1-4 and conserved regions were presented. Amino acid similarities and isoelectric point in HVRs Loop L1 were as follows: HVR1- 47. 2% (4.31), for HVR2- 60.5% (11.22), for HVR3- 59.9% (3.56), and for HVR4- 69% (9.66). For the comparison, the similarity between conservative regions in Loop L1 was (81.5%) with isoelectric point (26.84). Strains from type FAdV-4 indicated well-defined diversity as compared to the strains classified to the rest types. Their HVR1 was the shortest amongst the compared regions and spanned over the section of 62 aa. It indicated the lowest similarity to the rest of examined types in nucleotide and amino acid sequence. Isoelectric point of HVR1 was about (6.58) with average at (3.93).

**Table 4:** Nucleotide and amino acid position of the HVRs1-4 regions in the examined fragment of Loop L1 hexon gene of adenovirus strains classified to different types.

type	HVR1 Nucleotide Amino acid	HVR2 Nucleotide Amino acid	HVR3 Nucleotide Amino acid	HVR4 Nucleotide Amino acid	Length of examined region
FAdV-1/A AC_000014	49–240 17–80	241–288 81–96	334–426 112–142	481–498 161–166	519 173
FAdV-2/11/D KT862805 KT862812	49–243 17–81	244–291 82–97	337–429 113–143	484–501 162–167	522 174
FAdV-4/C HE608152	49–237 17–79	238–276 80–92	322–414 108–138	469–486 157–162	507 169
FAdV-5/B KC493646	49–252 17–84	253–300 85–100	346–438 116–146	493–504 165–170	531 177
FAdV-7/E KT862809	49–246 17–82	247–294 83–100	340–432 114–144	487–510 163–168	525 175
FAdV-8a/E KT862810	49–246 17–82	247–294 83–98	340–432 114–144	487–504 163–168	525 175
FAdV-8b/E KT862811	49–252 17–82	253–294 83–98	340–432 114–144	487–504 163–168	525 175

**Fig 2:**
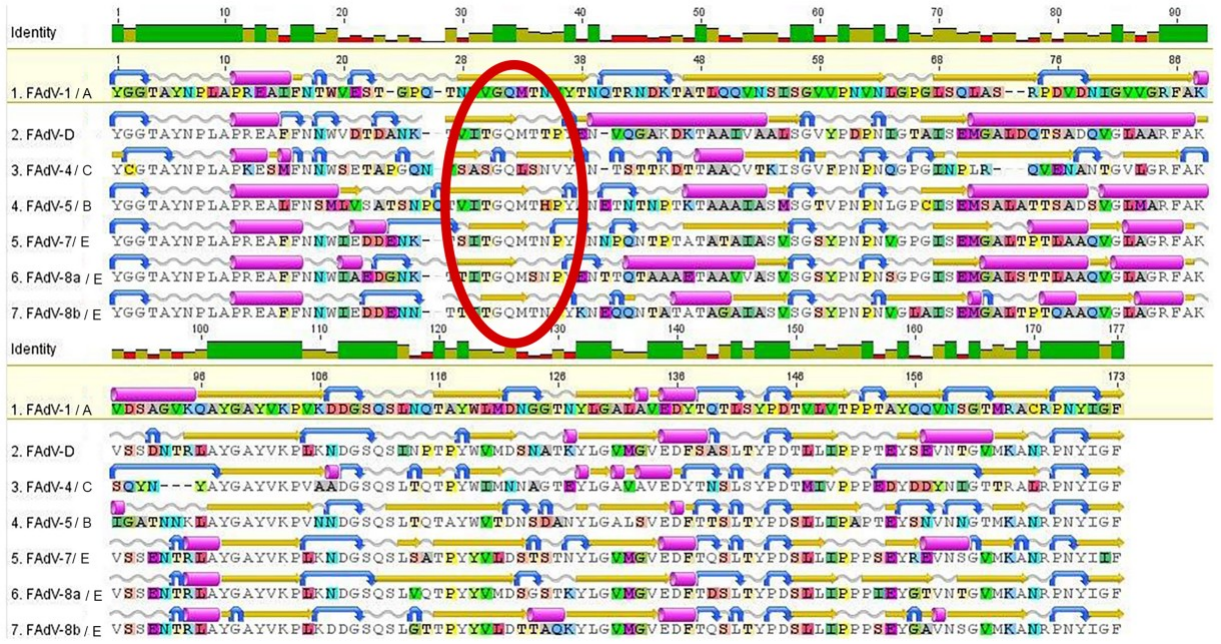
Annealing of consensus amino-acids sequences of the examined region HVRs1-4 Loop L1 of hexon gene of adenovirus strains/types. The location under the sequences indicate the predicted on the basic amino-acid sequence presence of tertiary structures: α-helix (pink), β-sheet (yellow), curve segment (blue), without structure definition (gray).

Analysis of theoretical tertiary structures which can be formed by examined sequences have been performed. As the base and model sequence for tertiary structure the reference strain FAdV-1/A (GenBank AF339914) sequence from NCBI was used. Consensus sequence HVRs1-4 of field adenovirus strains which belongs to type FAdV-1/A indicated high similarity of about (99.6%) with Loop L1 reference sequence with the differences only in two nucleotide in position: 98 nt and 223 nt. Amino acid sequences with indicated and selected tertiary structure were presented in Fig 2. Analysis of tertiary structure of protein hexon indicated that HVR1 created the loop L1 which is standing of hexon particle on the outside part of the virion. It is interesting, that we have found the peak of loop L1 is created by the characteristic amino acid sequence, strongly conservative in bird adenoviruses. This sequence GQMTN existed in examined strains from type FAdV-1/A, FAdV-7/E, and FAdV-8b/E. Type FAdV-2/11/D have the sequence GQMTT, and type FAdV-4/C sequence GQLSN with substitution M on L and S on T which are the substitutions functionally conservative. Type FAdV-5/B has GQMTH, and type FAdV-8a/E sequence GQMSN.

A hundred and thirty seven nucleotide DNA sequences of the examined strains were collected for this research. The sequences were compared to the sequences of reference FAdVs strains by the computer software and then compared to all others. The alignment of sequences was also performed by other authors [3, 13, 14, 15], and described very similar methods. Seven consensus nucleotide sequences were created: FAdV-1/A, 2/11/D, 4/C, 5/B, 7/E, 8a/E, and 8b/E and have been used during further analysis. The field strains formed five main branches representing FAdV-(A-E) species. FAdV-E branch was additionally divided into three sub-branches, with three types: FAdV-7/E, FAdV-8a/E, and FAdV-8b/E. On this basis, the adenovirus strains were classified into eight types representing five species FAdV-(A-E).

In the next step of the study, characteristics of sequences of FAdV strains were compared to the reference strains. Percentage of nucleotide similarity of the examined strains in the same type (intra-group) was between (89.7%) and (93.%). This was consistent with the results published by Kajan, 2013 [16]. At this time, clear differences appeared between strains classified to different FAdV types. Data confirmed correctness in classification of examined strains.

The analysis was based on properties of hexon gene, which is the biggest gene in adenovirus genome. Hexon gene has specific nature and structure with conserved and hypervariable regions HVR1-4, and is the object of most adenovirus studies based on taxonomy and characteristic antigenic properties [3, 13, 14, 17, 18, 19, 20]. It is very difficult or nearly impossible to conduct taxonomy studies based on conserved sequence, which is very similar and almost identical in all adenovirus types/species [3, 13, 14, 18]. That is why in this study Loop L1 HVRs1-4 region of hexon gene was used for analysis. This region can help in distinguishing the differences between strains of different types, and pointed out their diversity. In study conducted by Niczyporuk, 2015, the geographic analysis of adenovirus strains isolated in Poland based on loop L1 region of hexon gene have been described, and all the RSCU in HVRs1-4 were designated (data will be published in 2018).

Because of high sequence similarity between strains from FAdV-2/D and FAdV-11/D, it was impossible to discriminate between these two types and therefore 56 strains could not be assigned to any of these groups. To be in order with the ICTV classification system, the strains were described as species FAdV-2/11/D.

The percentage similarity between consensus sequences of different types was calculated and it was between (51.2%) for FAdV-2/11/D and FAdV-4, and (86.7%) for FAdV-7 and FAdV-8b. Type FAdV-4 was the most distant from other FAdVs and indicated multiple differences.

Alignment of consensus sequences clearly indicated the differences in Hypervariable Regions HVRs described by Raue, 2005 [21]. 4 HVRs in Loop L1 was identified as the regions of the highest sequence variability: HVR1 of about 191 bp long, HVR2 of about 50 bp long, HVR3 of about 90 bp, and HVR4 of about 18 bp long. The DNA sequences of HVRs are constant for every species, but there are major differences between FAdVs types.

Singh, 2015 indicated that trimer stability in TAdV-3 fibre head monomer has the surface area in trimer, and what can suggest, that fiber head from other adenovirus strains suggesting that the stability of the trimer are comparable. The melting temperatures virulent and avirulent forms of protein are 80°C at pH = 6 what can indicate high protein stability. Nasz and Adam, 2006 [22] indicated that adenovirus capsid amino acids are in symmetrical location in the inner and outer side with the icosahedral symmetry. Each hexon has six nearest neighboursand, and every hexon take part in the contstruction of three hexon rows. Every triangular facet participates in forming three vertices, and every facet has three nearest neighbouring facets.

Mutations in genes, their quantity and location can influence on the protein structure. In order to analysis this effect, nucleotide sequences were translated into amino acid sequences (aa), and then the resulting sequences of 176 amino acid long were analysed. As many authors suggest [4, 7, 12], mutations on the 1st and 2nd place of codon are the most important, because these mutations can influence on amino acid coding and subsequently change the structure and protein function. The theoretical tertiary structures of polypeptide chains conformed by the examined sequences were created. The examinations were based on the presence of structures (alfa-helix or beta-sheet), which were created by corresponding amino acids. The hexon protein of reference strain FAdV-1 (AF339914) served as the standard tertiary model of the hexon trimer. Amino acid consensus sequence of field adenovirus strains was fully comparable to the amino acid sequence of reference structure. Root of the protein is created by the highly conservative regions, almost identical in every adenovirus types isolated from birds, horses, cattle, and human [23]. The differences are focused mainly in Loop L1, and Loop L2. Loop L1, together with HVR1-4 regions is the longest loop in protein with complicated folding [24]. It also serves as the location of specific receptors [3, 22, 25].

Analysis of sequences: GQMTN, GQMTT, GQLSN, GQMTH, and GQMSN in examined HVRs1-4 Loop L1 region of hexon gene are visibly conservative, and it is surrounded by strong hyper variable regions which create bridge pile in Loop L1. The degree of behavior of this sequence among adenoviruses existed in birds indicated, that most probably to come true not precised yet important function. Examinations on the regions which are responsible for the antibodies binding [14] indicated, that this bridge pile are responsible for it [14]. All mutations which are situated in HVR1-4 region, can led to avoid host mechanisms of immunity, and face modifications of this regions can led to create vectors to clinic treatment. Hyper variable regions indicates high differentiation between adenovirus species/types and between the adenovirus strains which can infect different hosts. Simultaneously comparison study on amino acid protein hexon sequences of adenovirus strains from different species conducted by Rux, 2003 indicated that amino acid sequences of HVR1 are specific for exact adenovirus host. During the study the medium isoelectric point was measured and was indicated as 4.48.

HVR1 region forms the structure called „hairpin” [26, 27, 28] which has sequence GQMT at the end, which was in examined strains classified to types FAdV-1/A, 2/11/D, 5/B, 7/E, and 8b/E, with the exception of type 4/C, which had the sequence GQLS, and strains from type 8a/E with sequence GQMS. Analysis of the HVR1 structure of the strains suggests the presence of β-sheet structure which is created by the „hairpin”. This structure was found in all FAdV types. HVR3, similarly to HVR1, also forms „hairpin” structure which is forming above protein surface. HVR1 and HVR3 are positioned close to each other in shape of V. The HVR5 (sequence not in the Loop 1) is positioned between these V-structure arms. Study by Pichla-Gollon, 2007 indicated that the side of hairpin structure of HVR1 is the main region for the neutralizing antibody binding.

## Conclusions

In conclusion, 137 adenovirus strains have been isolated from infected chickens. The DNA of all strains have been isolated, and sequencing have been performed. In a homogeneous population of adenovirus strains the differences highlighted the importance data in examined HVRs1-4 region. Obtained sequences can be helpful to better understand the antigenic receptors of viruses. This results encourage us to target this region for futures studies. Indeed, HVRs1-4 examined sequences highlighted the most interesting regions: GQMTN, GQMTT, GQLSN, GQMTH, and GQMS. This allowed us to analyze over 137 adenovirus strain sequences, and their descriptive classification seems to be relevant for further analysis among adenovirus type/species. This could lead to better understanding of the heterogeneous etiology of the diseases caused by adenovirus species, as well as provides a new preventive strategies based on this data.

This is the first study conducted on HVRs1-4 loop L1 tertiary structure sequences obtained from adenovirus field strains in Poland. Because the mechanism of pathogenicity are not clear enough, and adenovirus infections are the reason of economic losses in poultry flocks industry. This study are needed and can help to better understand the mechanism of pathogenicity and the molecular investigation will be improved and continued.
